# Indirect assessment of biomass accumulation in a wastewater-based *Chlorella vulgaris* photobioreactor by pH variation

**DOI:** 10.1038/s41598-021-98634-0

**Published:** 2021-09-30

**Authors:** Francesca Nyega Otim, I-Ru Chen, Ochan Otim

**Affiliations:** 1 Department of Anthropology, University of California, Davis, 1 Shields Ave, Davis, CA 95616 USA; 2 Department of Humanities and Sciences, University of California, Los Angeles, 10960 Wilshire Boulevard, Los Angeles, CA 90024 USA; 3 Environmental Monitoring Division, City of Los Angeles, 12000 Vista Del Mar, Playa Del Rey, CA 90293 USA

**Keywords:** Environmental sciences, Pollution remediation

## Abstract

Algae bloom in coastal waters is partly supported by residual nutrients in treated wastewater (WW) released from coastally located treatment plants. In response, a *Chlorella vulgaris*-based photobioreactor was recently proposed for lowering nutrient levels in WW prior to release. However, the solution requires maintaining biomass accumulation to within a photobioreactor capacity for optimum operation. For high density *Chlorella vulgaris* suspensions, this is easily done by monitoring turbidity increase, a property directly related to biomass accumulation. For low density suspensions however, direct turbidity measurement would require a cumbersome process of concentrating large volumes of *Chlorella vulgaris* suspensions. Here, we demonstrate that by measuring pH of the suspensions, turbidity (T) can be estimated indirectly by the following wastewater-dependent expression: pH = *a*T + pH_0_, hence avoiding the need to concentrate large volumes. The term pH_0_ is the initial pH of the suspensions and *a*, a wastewater-dependent constant, can be computed independently from *a* = − 0.0061*pH_0_ + 0.052. In the event %WW is unknown, the following wastewater-independent Gaussian expression can be used to estimate T: pH = 8.71*exp(− [(T − 250)^2^]/[2*1.26E05]). These three equations should offer an avenue for monitoring the turbidity of dilute *Chlorella vulgaris* suspensions in large, stagnant municipal *Chlorella vulgaris*-based wastewater treatment system via pH measurements.

## Introduction

Harmful algae and eutrophication of large water bodies are linked to environmental degradation which occasionally manifests graphically as fish die-offs and seabirds poisoning in coastal waters^1–3^. The algae, usually occurring as blooms, are supported in part by increased amounts of nutrients entering aquatic environment from anthropogenic sources such as agricultural runoff, stormwater and coastal municipal wastewater treatment plants^4–7^. The consequence of algal blooms on sensitive organisms, those that depend on the water bodies for survival, are many, a few of which are oxygen depletion^8^, sunlight blockage^9^, and increased secretion of toxic chemicals into the surrounding marine environment^10^. As a result, coastal municipalities devote a considerable amount of resources in attempts to mitigate eutrophication, some of which—in the USA—are mandated by the National Pollutant Discharge Elimination System (NPDES) permits issued to parties responsible for discharging nutrients into the environment^11,12^.

Around the world, several solutions have been adopted for reducing levels of nutrients in treated wastewater. A few of these are chemical in nature and include precipitating and separating out nutrients from wastewater before release^13^. Others are biological in nature (reviewed by^14^) which include the deployment of microalgae as a manageable solution^15^. To the latter was added *Chlorella vulgaris* (*C. vulgaris*) in 2019 as a specific solution for not only reducing nutrients in secondary-treated municipal wastewater, but also as one promising means of sequestering atmospheric or flue gas-derived CO_2_ simultaneously^7,16^. *C. vulgaris*, a small freshwater microalga (5–50 µm), carries a negative surface electric charge and forms a stable suspension as a consequence ^17^. When the external supply of CO_2_ is limited, the pH of a *C. vulgaris* suspension will rise with time due to the consumption of CO_2_ during photosynthesis^17,18^. The said pH increase occurs in spite of the basal pH variation caused by nitrogen uptake during cell growth (i.e., when cells used NH_4_^+^ ion in metabolism, H^+^ extrusion occurs^19–21^, hence the pH of the surrounding goes down; but when cells used NO_3_^-^ ion instead, the pH of the surrounding goes up^22^).

A successful implementation of the *C. vulgaris* approach requires monitoring and maintaining microalgae density to within the capacity of a photobioreactor. This is easily accomplished in high density microalgae suspensions by monitoring increase in turbidity, or by measuring optical density at some appropriate pigment absorption wavelength. When density is low however, the accuracy and the precision of such optically-based measurements are affected by light intensity, nutrients supply and type, source of organic carbon, and the fact that microalgae cells must cross the optical path of a detector by slow natural diffusion to be recorded as present. Here, we show that the temporal and parallel variations of both turbidity and pH in a wastewater-based *C. vulgaris* photobioreactor could be exploited by regression to monitor turbidity, and hence biomass accumulation indirectly. This approach is premised on the known fact that the turbidity and the pH of a *C. vulgaris* suspension rise with time naturally^7,18^ and the fact that a pH-based measurement is not only convenient and straightforward, it is cheaper when compared to other cell measuring methods.

## Materials and methods

All containers used in this study were disinfected with 3% aqueous hydrogen peroxide solution and rinsed with distilled water.

### Wastewater source

Secondary-treated wastewater was acquired from the Hyperion Water Reclamation Plant (HWRP, City of Los Angeles, CA, USA). Its parameters were determined as described elsewhere^6^.

### Microalgae

The initial stock of *C. vulgaris* was purchased from Carolina Biological Supply (Burlington, NC) and used as delivered without measuring the concentration. A laboratory stock of the microalgae were maintained in a 30-L distilled water (DH_2_O)-open photobioreactor equipped with a capacity to boost CO_2_ level in microalgae culture automatically when pH > 7.4. A Guillard’s (F/2) nutrient concoction (20 mL) from the *C. vulgaris* vendor was added to the laboratory stock every 7 days for the duration of data collection (35 days).

### Experimental

Experiments were performed as described earlier^7^. In brief, seven 310-mL wastewater-to-distilled water ratio (WW/DH_2_O) photobioreactors were set up in duplicates in open Erlenmeyer flasks as follows (v/v): 0/300, 50/250, 100/200, 150/150, 200/100, 250/50, 300/0. Into each was added 10 mL of laboratory stock *C. vulgaris* and allowed to grow without stirring under an alternating cycle of artificial white light (rated at 6500 K) for 16 h and darkness for 8 h. The experiment ended when *C. vulgaris* turned brown (35 days). Yield was monitored by changes in turbidity (NTU) using NUL-231 Turbidity Logger Sensor coupled to a USB-200 USB Module (Rochester, NY, USA).

An integrated pH monitoring system (Doctors Foster and Smith, Rhinelander, WI, USA) was used to measure pH of microalgae suspensions. The components of the system included a Pinpoint pH electrode with a 00.00 pH Unit resolution over a pH range of 1.00–14.00 (America Marine Inc., Ridgefield, CT, USA). On each day of pH data collection, a two-point electrode calibration was carried out at pH 7.00 and at pH 10.00 using certified reference buffer solutions (Fisher Chemicals, Fair Lawn, NJ, USA) prior to measuring the pH of samples.

The rate at which *C. vulgaris* consumed nutrients and CO_2_ during incubation was previously done at an empirical level, the results of which showed the lowest growth rates occuring (1) in a photobioreactor configured to be open to the atmosphere with no additional CO_2_ supply, or (2) in the best performing photobioreactor configuration (i.e., open with additional CO_2_ supply), but when additional nutrients were not supplied^7^. These results were assumed in the current study.

### Correlation verification

Hierarchical Clustering on Principal Components (HCPC)^23^ was used to verify correlation between pH and turbidity. HCPC is a statistical technique which employs principal component analysis (PCA) to reduce multidimensional data to a few with the most important information before handing over the ‘cleaned up’ data to the cluster analysis (CA) technique for detecting similarities. Verification here meant similar samples would not only group together, but would do so in a temporal order. *Factoshiny* software package running within *RStudio* was used for HCPC analysis^23–25^. General statistical analyses were carried out using PAleontological STatistics (PAST) software package^26^.

## Results and discussion

### pH–turbidity relationship

For reference, the constituents of interest in the secondary-treated wastewater used in the current study are provided in Table 1.

**Table 1:** Composition of secondary-treated wastewater used.

Analyte	Amount per L
Antimony	2.75 µg
Arsenic	2.30 µg
Beryllium	0.03 µg
Boron	541 µg
Cadmium	0.05 µg
Chloride	3.03E + 5 µg
Copper	13.4 µg
Mercury	2.39E-3 µg
Nickel	6.52 µg
Silver	0.061 µg
Thallium	0.02 µg
Zinc	14.8 µg
Selenium	0.52 µg
Carbonaceous biochemical oxygen demand	5 mg
Biological oxygen demand	12 mg
Total suspended solids	9.7 mg
Total dissolved solids	768 mg
Total organic carbon	19.1 mg
Total Kjeldahl nitrogen	47.8 mg
Total phosphate	2.82 mg
Ammonia as nitrogen	47.0 mg
Nitrate as nitrogen	not detected
Nitrite as nitrogen	0.32 mg

Data available at http://ciwqs.waterboards.ca.gov/ciwqs/readOnly/PublicReportEsmrAtGlanceServlet?reportID=2&isDrilldown=true&documentID=1893752.

**Figure 1:**
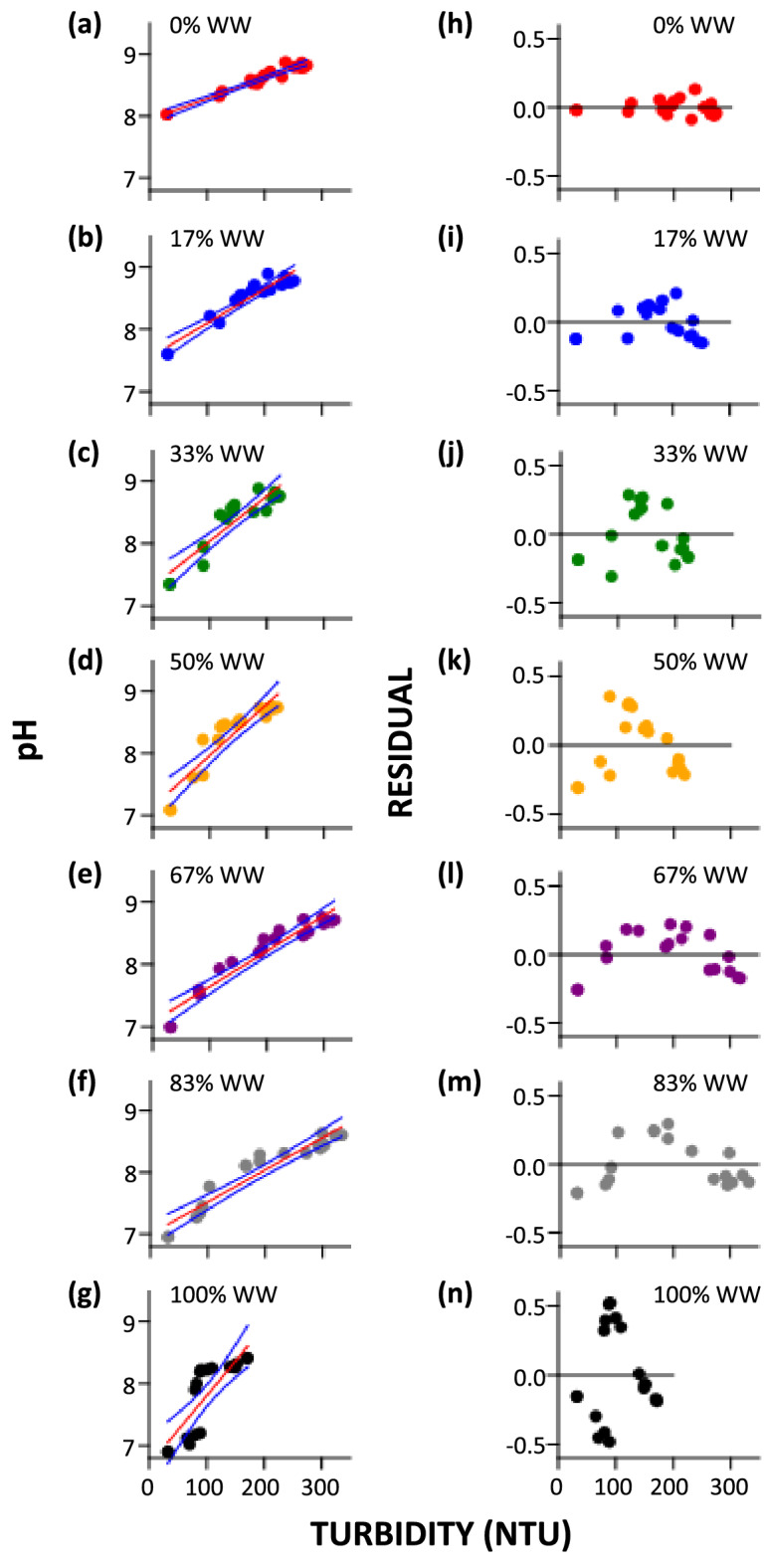
Scatter plots of pH changes as a function of turbidity (**a**)–(**g**) and associated residual plots (**h**)–(**n**) under different WW/DH_2_O ratios. pH and Residual pairs (**a**) and (**h**) are for the 0%WW/100%DH_2_O ratio, (**b**) and (**i**) pair: 17%/83%, (**c**) and (**j**): 33%/67%, (**d**) and (**k**): 50%/50%, (**e**) and (**l**): 67%/33%, (**f**) and (**m**): 83%/17%, and (**g**) and (**n**): 100%WW/0%DH_2_O ratio.

To relate the pH of a microalgae suspension, a relatively easy quantity to measure, to the turbidity of the suspension, a surrogate for biomass increase but challenging to measure in low density microalgae suspension, the pH and the turbidity of seven different duplicates of secondary-treated wastewater dilutions containing the same amount of *C. vulgaris* were followed over the course of a 35-day period. Results show that a higher turbidity is associated temporally with a higher pH, irrespective of wastewater dilution (Fig. 1), and at each dilution, the relationship can be modeled by a linear expression of the form

1 $$\mathrm{pH}=a\mathrm{T}+{\mathrm{pH}}_{0}$$

where *a* and pH_0_ are both wastewater-dependent, and are changes in pH per unit change in T and the initial pH predicted by regression, respectively (Fig. 1a–g). This linear relationship is supported by the lack of pattern in the associated residual plots (Fig. 1h–n; except perhaps for the 67% and the 83% WW dilutions). It is also supported by the regression statistics (Table 2) which shows (1) by the values of *r* that the strength and direction of the linear relationship between pH and turbidity is strong and positive, (2) by the narrower 95% confidence intervals obtained by bootstrap resampling 1999 times that perhaps outliers were not important in data fitting, and (3) by standard errors of the mean which show that our data may possibly represent similar data. The probabilities of alternative explanations to each of these are extremely low (last column of Table 2).

**Table 2:** pH versus turbidity (T) least squares regression (pH = *a*T + pH_0_**)** statistics for photobioreactors with different proportion of wastewater (%WW) in distilled water (DH_2_O).

%WW	Slope *a*	SE *a*	Intercept pH_0_	SE pH_0_	95% Bootstrapped CI (*N* = 1999)		*r*	*p*(uncorr.)
					Slope *a*	Intercept pH_0_		
0	3.34E−03	1.83E−04	7.95	3.75E−02	(3.04E−03, 3.75E−03)	(7.86, 7.98)	0.977	4.11E−12
17	5.46E−03	4.50E−04	7.56	8.22E−02	(4.68E−03, 7.17E−03)	(7.21, 7.66)	0.950	1.75E−09
33	7.21E−03	8.00E−04	7.32	1.29E−01	(5.97E−03, 9.58E−03)	(6.89, 7.49)	0.914	1.14E−07
50	8.26E−03	9.14E−04	7.14	1.37E−01	(6.54E−03, 1.13E−02)	(6.61, 7.39)	0.914	1.10E−02
67	5.67E−03	4.15E−04*	7.08	8.99E−02	(4.81E−03, 7.07E−03)	(6.74, 7.25)	0.960	3.12E−10
83	5.22E−03	4.18E−04*	7.00	9.13E−02	(4.51E−03, 6.29E−03)	(6.72, 7.14)	0.952	1.18E−09
100	1.11E−02	1.94E−03	6.69	2.15E−01	(8.64E−03, 1.45E−02)	(6.19, 6.99)	0.821	3.02E−05

CI, confidence interval; SE, standard error; *p*(uncorr.), probability that pH and turbidity are uncorrelated.

*Predicted values are 8.40E−04 and 8.9E−04, serially.

**Figure 2:**
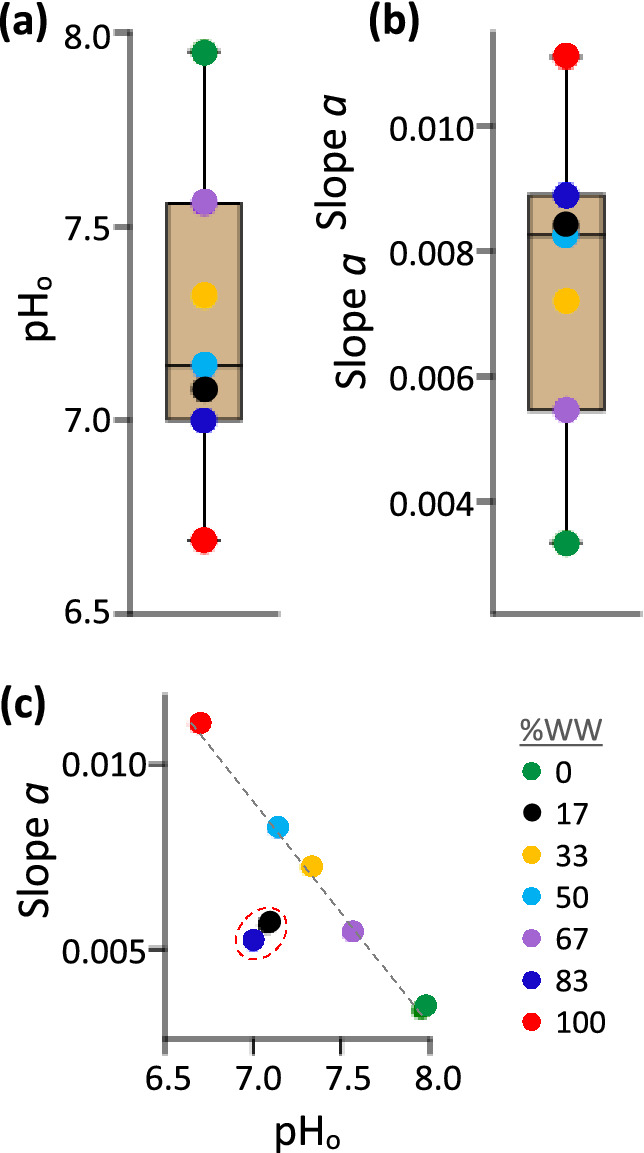
Box plots showing the distribution of (**a**) pH_0_, the initial pH values derived from Eq. (1), and (**b**) the accompanying slope *a*. (**c**) The linear relationship of *a* to pH_0_, expressed as *a* = − (0.0061* pH_0_) + 0.052. Expected *a* values for the 67 and 83% WW were used in (**b**); the observed values for 67 and 83% WW are encircled in (**c**) to highlight this need.

To adopt this approach of monitoring biomass accumulation via turbidity estimates from pH, prior knowledge of the wastewater-dependent constant *a* is required. By a plot of *a* versus pH_0_ (second and forth columns, Table 2), we found that *a* and pH_0_ are inversely related (compare Fig. 2a and b, a trend summarized in Fig. 2c)and can be expressed as

2 $$a= -0.0061*{\mathrm{pH}}_{0}+0.052$$

In practice, experimentally observed initial pH could be used in lieu of pH_0_ in Eq. (2) to determine *a*. This substitution is permissible because no significant difference was observed between the scores of *regression* pH_0_ (mean = 7.25, standard deviation = 0.41) and the *observed* pH_0_ (mean = 7.27, standard deviation = 0.42); *t*(12) = 0.1165, *p* (same mean) = 0.909.

Overall, Eqs. (1) and (2) should provide a means of monitoring *C. vulgaris* biomass accumulation at any %WW without relying on turbidity measurements. A verification of this concept follows.

### Verify correlation by HCPC

**Figure 3:**
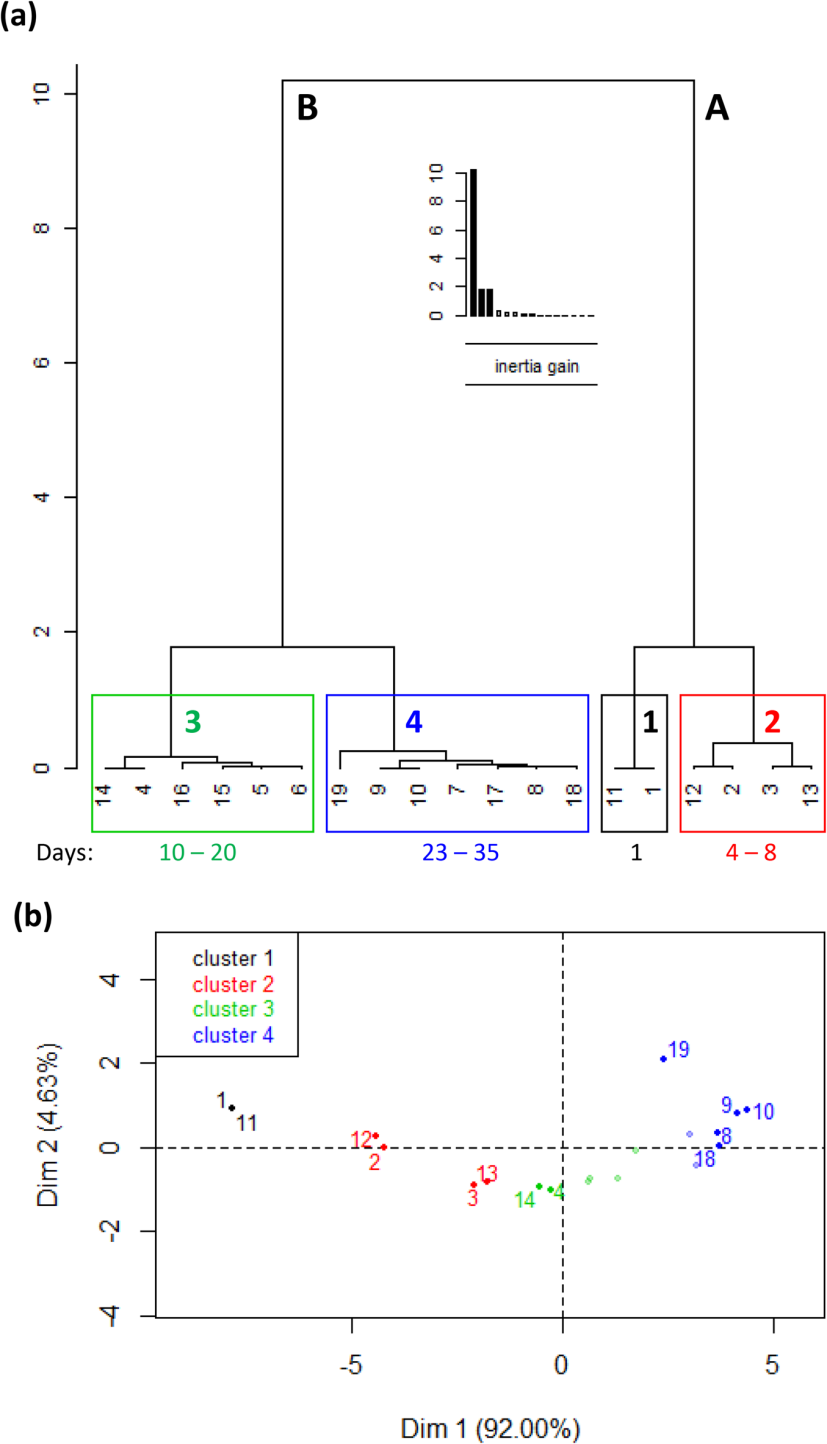
HCPC results showing the coalescing of 19 × 7 multivariate pH and turbidity data into four distinct groupings. (**a**) Dendrogram showing hierarchical similarity of measurements as a function of the length of incubation. (**b**) Scatter plot of scores of the measurements in (**a**) along the most important HCPC dimensions: Dim and Dim 2 (explaining 92.00% and 4.63% of variance, respectively). The natural groupings of scores are labeled or color-coded as cluster 1 (black), cluster 2 (red), cluster 3 (green) and cluster 4 (blue).

To provide additional level of assurance, HCPC was used to analyze the same data with the expectation that a temporal order of data association would be observed. The results, displayed in Fig. 3, show that there was indeed a temporal order of association. The data are clearly separated by HCPC, not only into two very distinct groupings (Fig. 3a) based on whether they were collected earlier (**A**: 1–8 days) or later (**B**: 10–35 days), but each grouping was additionally separate into two temporal clusters. Within the grouping labeled **A**, the cluster labeled 1 is made of individuals 1 and 11 characterized by low values of both pH and turbidity in all the wastewater setups, save the 100% WW (i.e., 0%, 17%, 33%, 50%, 67% and 83%WW). These two individuals (1 and 11) were measurements acquired in duplicate samples at the beginning of the current study when microalgae growth had not settled into their new medium (one the microalgae were not supplied in). Similarly, the membership of the cluster labeled 2 in grouping **A** is made up of measurements on two duplicate samples 2 and 12, and 3 and 13, the data of which were acquired 4–8 days after the start of incubation. This cluster is characterized by low values for the pH in 83% and 100%WW, and turbidity in 67% and 83%WW, meaning that at the onset of incubation, growth started in all but in these two highly concentrated wastewater-based *Chlorella vulgaris* suspensions. The latter was expected^7^.

In the grouping labeled **B**, the cluster labeled 3 is made of data from sample pairs 4 and 14, 5 and 15, and 6 and 16 which are characterized by pH and turbidity values not significantly different from their mean values. Data in this cluster were collected midway towards the end of the study, thus verifying the observed temporal trends. The cluster labeled 4 is made up of data pairs such as 7 and 17, 8 and 18, and 9 and 19 which are characterized by high values for the two variables in all suspensions. This was expected since these were terminal sets of data.

By these temporal sequence of grouping and clustering (**A** followed by **B,** and cluster 1 followed by clusters 2, 3 and 4), it is reasonable to conclude that results of HCPC analysis supports the results of descriptive analysis provided at the beginning of this Results and Discussion section. Assurance is therefore provided in support of monitoring biomass accumulation in suspensions of *C. vulgaris* in treated wastewater by the indirect approach proposed.

### Limit of pH-turbidity linear relationship

**Figure 4:**
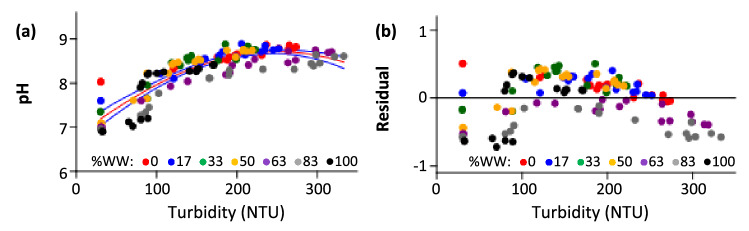
Integrating 126 bivariate data pooled from seven WW proportions used in Fig. 1 into single planes. (**a**) Scatter plot showing lack of linearity between pH and turbidity overall. Data can be model by the equation pH = 8.71*exp(− [(T − 250)^2^]/[2*1.26E05]); 95% confidence interval limits included as blue lines. (**b**) Associated residual plot showing lack of randomness expected from a linear relationship between pH and turbidity at all %WW. Data corresponding to each WW proportion is color-coded.

This study shows that at each %WW, an increase in turbidity correlates with an increase in pH (Fig. 1) and that an overall increase in %WW correlates with an increase in *a* (the amount by which pH changes per unit change in turbidity, Table 2). These observations appear to negate the need to specify WW proportion when monitoring biomass accumulation in a photobioreactor via pH measurement. That is, the linear relationship between pH and turbidity shown here may appear universal. To determine whether this is actually valid, the 126 bivariate pH-turbidity measurements collected from the seven sets of WW/DH_2_O dilutions were plotted on the same plane along with their associated residuals on a similar plane (Fig. 4). From the pattern, a higher turbidity is indeed associated with a higher pH universally. However, the relationship is not linear (Fig. 4a), an observation confirmed by the existence of a curve pattern in a residual plot when linearity was assumed (Fig. 4b). This finding simply means there is a limit to the linear pH-turbidity relationship outside a specifically defined %WW values. By this finding, Eqs. (1) and (2) should therefore be used only when the initial amount of wastewater in the microalgae suspension is known.

### Alternatives to linear model

As shown in Fig. 4, %WW in a photobioreactor should be specified when monitoring *C. vulgaris* biomass accumulation via the linear relationship between pH and turbidity by our proposal. To remove the need to specify %WW, nine alternative growth models were tested for a close fit with the same 126 bivariate measurements. A good fit was assessed by grading each model on the Akaike information criterion (AIC)^27^ which quantifies the amount of information that would be lost if a model was used. The smaller the AIC value in comparative terms, the better the predictive value of the model. The test results show that the Gaussian model Eq. (3) (AIC: 14.25) was a better fit here

3 $$pH={ae}^{- \frac{{(T-b}^{2})}{{2c}^{2}}}$$

In Eq. (3), *a*, the height of the curve, occurs at pH 8.71, and *b*, the position of peak center, and *c*^2^, the variance, are 250 NTU and 1.26, respectively. The curve fitting is shown in Fig. 4a (red curve), along with the associated 95% confidence interval limits (blue curves).

The second best growth model was the logistic model (14.86), followed by Gompertz (14.93), von Bertalanffy (15.01), Hill’s sigmoidal (15.49), allometric (15.91), Michaelis–Menten (16.33), linear (17.71) and lastly, the exponential model (19.82).

### Limitation of this study

This study has three limitations. First, by not stirring the algal suspensions during the 35 days of incubation, which was meant to mimic a commonly seen stagnant algae pool, factors such as autoflocculation^17,28^, alkalinity-induced aggregation^29^, and aggregation between microalgae and bacteria^30^ were unaccounted for. Secondly, studies using water sources native to the environment such as the sea/ocean, inland lakes or river for diluting wastewater would have approximated better the targets of this study. Such sources are the receiving water bodies for treated wastewater. And third, because the secondary treated wastewater was not sterilization, albeit disinfected with sodium hypochlorite before use, the possibility of other microbes being involved in the observed turbidity change is a possibility. In our opinion, this is unlikely given the rigor of wastewater treatment at the Hyperion Water Reclamation Plant mandated by public health concerns and the intended use of the treatment product^31^.

## Conclusions

In the present study, we have shown that pH, a homogenous solution property, offers a predictable and a straightforward alternative to measuring the turbidity of a low density microalgae suspension. This need arises because the reliability of direct turbidity measurements in such suspension is low due to reliance on light scattering for measurement which, in turn, depends on the actual count of particulate algae crossing the optical path of the measuring instrument.

## Data Availability

All data are available on request from the corresponding author.
